# Supplementary material for: Active site specificity profiling datasets of matrix metalloproteinases (MMPs) 1, 2, 3, 7, 8, 9, 12, 13 and 14
Source: Data Brief. 2016 Feb 22;7:299–310. doi: 10.1016/j.dib.2016.02.036 (PMC4777984; doi:10.1016/j.dib.2016.02.036)
Supplement: Supplementary file 10 — Supplementary material [file mmc10.zip › WebPICS_hMMP8_T_1%/P3.html]

 

PICS results


|  |  |
| --- | --- |
| **P3\_A**  44 in 335 sites   13.1 %    effects > 10 perc. pnts.  (vice-versa in brackets)  P2\_L: 12.1 (10.4) |  |
| **P3\_P**  68 in 335 sites   20.3 %    effects > 10 perc. pnts.  (vice-versa in brackets)  P2\_E: 10.4 (24.5) |  |
| **P3\_T**  19 in 335 sites   5.7 %    effects > 10 perc. pnts.  (vice-versa in brackets)  P2prime\_H: 12.2 (19.3) |  |
| **P3\_V**  33 in 335 sites   9.9 %    effects > 10 perc. pnts.  (vice-versa in brackets)  P2\_S: 10.1 (12.3)   P3prime\_S: 10.8 (10.1) |  |
